# Wnt-signaling enhances neural crest migration of melanoma cells and induces an invasive phenotype

**DOI:** 10.1186/s12943-018-0773-5

**Published:** 2018-02-17

**Authors:** Tobias Sinnberg, Mitchell P. Levesque, Jelena Krochmann, Phil F. Cheng, Kristian Ikenberg, Francisco Meraz-Torres, Heike Niessner, Claus Garbe, Christian Busch

**Affiliations:** 1 Center for Dermatooncology, Department of Dermatology, University Hospital Tübingen, University of Tübingen, Liebermeisterstr.25, 72076 Tübingen, Germany; 2 Department of Dermatology, Universitaets Spital Zürich, Gloriastrasse 31, 8091 Zürich, Switzerland; 3 Institute of Clinical Pathology, University Hospital Zürich, Schmelzbergstrasse 12, 8091 Zürich, Switzerland; 4 Dermateam, Bankstrasse 4, 8400 Winterthur, Switzerland

**Keywords:** Melanoma, Epithelial mesenchymal transition, Wnt3a, β-catenin, Metastasis, Invasion

## Abstract

**Background:**

During embryonic development Wnt family members and bone morphogenetic proteins (BMPs) cooperatively induce epithelial-mesenchymal transition (EMT) in the neural crest. Wnt and BMPs are reactivated during malignant transformation in melanoma. We previously demonstrated that the BMP-antagonist noggin blocked the EMT phenotype of melanoma cells in the neural crest and malignant invasion of melanoma cells in the chick embryo; vice-versa, malignant invasion was induced in human melanocytes in vivo by pre-treatment with BMP-2.

**Results:**

Although there are conflicting results in the literature about the role of β-catenin for invasion of melanoma cells, we found Wnt/β-catenin signaling to be analogously important for the EMT-like phenotype of human metastatic melanoma cells in the neural crest and during invasion: β-catenin was frequently expressed at the invasive front of human primary melanomas and Wnt3a expression was inversely correlated with survival of melanoma patients. Accordingly, cytoplasmic β-catenin levels were increased during invasion of melanoma cells in the rhombencephalon of the chick embryo. Fibroblast derived Wnt3a reduced melanoma cell adhesion and enhanced migration, while the β-catenin inhibitor PKF115–584 increased adhesion and reduced migration in vitro and in the chick embryonic neural crest environment in vivo. Similarly, knockdown of β-catenin impaired intradermal melanoma cell invasion and PKF115–584 efficiently reduced liver metastasis in a chick chorioallantoic membrane model. Our observations were accompanied by specific alterations in gene expression which are linked to overall survival of melanoma patients.

**Conclusion:**

We present a novel role for Wnt-signaling in neural crest like melanoma cell invasion and metastasis, stressing the crucial role of embryonic EMT-inducing neural crest signaling for the spreading of malignant melanoma.

**Electronic supplementary material:**

The online version of this article (10.1186/s12943-018-0773-5) contains supplementary material, which is available to authorized users.

## Background

Melanocytes are derived from the embryonic neural crest. During and after closure of the neural tube in the trunk of the embryo, the neural crest is induced by an intricate crosstalk between bone morphogenetic proteins (BMPs) and Wnt-signaling [1–4], which could be reproduced in human induced pluripotent stem cells in vitro [5]. In the course of this process, neural crest cells undergo morphological changes accompanied by an altered adhesion and migration capacity. A corresponding morphological transformation of chick embryonic lens epithelial cells was designated as epithelial-mesenchymal transition (EMT) [6]. After induction, neural crest cells migrate along their designated medial and lateral pathways. Neural crest cells following the medial pathway form spinal ganglia and the autonomic ganglia of the sympathetic chain. The neural crest-derived progenitors of melanocytes (melanoblasts) migrate along the lateral pathway and colonize the epidermis [7]. During invasion of the epidermal/dermal layer of the skin melanoma cells (arising from melanoblasts or melanocytes in the epidermis) morphologically pass through an EMT-like program and thus recapitulate their former neural crest cell specific migratory capacities.

In previous experiments, we observed that human SKMEL28 [8, 9] and mouse B16-F1 melanoma cells [10] spontaneously integrate into the neural crest and resume a physiological neural crest cell migration after transplantation into the neural tube of the chick embryo. In contrast to melanoma cells, neurospheres generated from adult mouse subventricular zone (SVZ) stem cells integrate into the neural crest and perform neural crest cell migration only after pre-treatment with bone morphogenetic protein-2 (BMP-2) [11]. BMPs are constitutively expressed in melanoma cells [12]. In line, we showed that physiological neural crest migration (after transplantation into the neural tube) and invasive growth (after transplantation into the optic cup of the chick embryo) of melanoma cells can be ablated by the BMP-antagonist noggin [13, 14] and that vice-versa malignant invasion can be induced by pre-treatment with BMP-2 in non-transformed human melanocytes [15] clearly demonstrating that embryonic neural crest signaling pathways play important roles in the invasive growth of melanoma cells in vivo.

In a second approach, we demonstrated that the embryonic transcription factor β-catenin is frequently activated during melanomagenesis via non-mutational alterations like reduced expression levels of casein kinase 1α resulting in protein stabilization of the central Wnt-signaling player β-catenin [16]. Furthermore, we showed in vitro a pro-migratory and pro-invasive function of β-catenin in melanoma cells implanted into human organotypic skin reconstructs [17] which seems context dependent due to controversial reports in the literature. In line with a pro-invasive function of neural crest signaling pathways, it was reported that leflunomide, an inhibitor of dihydroorotate dehydrogenase, leads to an abrogation of neural crest development in zebrafish and decreases melanoma growth in vitro and in mice. Leflunomide exerts these effects by an inhibition of neural crest development [18], also suggesting a close connection between developmental pathways and melanomagenesis. Leflunomide is an agonist of the ligand-activated transcription factor aryl hydrocarbon receptor (AhR) and activation by leflunomide leads to a dysregulation of Wnt signaling not least because its function as ligand-dependent E3 ubiquitin ligase in the β-catenin degradation pathway [19–21]. This was shown to have a tumor suppressing function in intestinal cancer [22]. However, the peculiarity of the canonical Wnt-signaling in the context of melanoma has produced conflicting theses about the correlation of the expression levels of β-catenin and the progression of the disease [23, 24], which shows that the role of β-catenin in melanoma metastasis remains largely unclear. This was nicely summarized by Weeraratna et al. [25]. Some studies indicated that β-catenin suppresses invasion of melanoma cells and that loss of β-catenin predicts a poor survival rate in melanoma patients [26–29]. Other studies showed that increased abundance or stabilization of β-catenin leads to increased melanoma metastasis, both in vitro and in vivo [30–34]. However, Wnt ligands do play a role in melanoma cell invasion. Consequently, Wnt5a promoted melanoma cell invasion and polarized protein localization by depalmitoylation of the melanoma cell adhesion molecule (MCAM) at cysteine 590 [35]. Interestingly, Wnt5a does not exlusively mediate its effects on melanoma cells via a non-canonical, protein kinase C (PKC) dependent Wnt signaling pathway [36, 37], but also stimulates β-catenin transcriptional activity during Wnt5a-mediated melanoma invasion and metastasis [33]. Lastly, it was demonstrated that in addition to driving melanomagenesis and invasion of melanoma cells, intrinsic β-catenin signaling prevents anti-tumor immunity, leading to T-cell exclusion and resistance to anti-PD-L1/anti-CTLA-4 monoclonal antibody therapy in melanoma mouse models [38], which could have great clinical significance.

Going back to embryologic neural crest induction, we asked in this project whether the Wnt3a/β-catenin signaling pathway was also directly involved in melanoma cell adhesion/migration in vitro and in vivo in an embryonic micro-environment of the neural crest. To this end we used in vivo chick embyo models including the experimental growth of melanoma brain [15, 39, 40] and liver metastasis [41, 42] along with in vitro models.

## Methods

### Cell lines, cell culture, and generation of Wnt3a-conditioned medium

The following human metastatic melanoma cell lines were used in this study: SKMEL28, A375, BLM, SKMEL19 and. 451Lu. Melanoma cells were cultivated in HEPES-buffered, 2 mM L-Glutamin containing RPMI1640-medium (Gibco/ Life Technologies, Darmstadt, Germany), which was supplemented with 10% fetal calf serum (Biochrom, Berlin, Germany) and 100 U ml^− 1^ Pen-Strep (Gibco/ Life Technologies, Darmstadt, Germany). All cultures were maintained at 37 °C in a 95% air / 5% CO_2_ atmosphere at 85% humidity. For generation of Wnt3a-conditioned medium (Wnt3a-CM), NIH3T3 murine fibroblasts constitutively over-expressing Wnt3a (kindly gifted by Prof. Michael Schwarz, Institute of Toxicology, University of Tuebingen, Germany) were cultured in DMEM-medium (Life Technologies, Darmstadt, Germany) supplemented with 1% FCS for 48 h. NIH3T3 conditioned medium (3T3-CM) served as control.

### Transcriptome analysis of SKMEL28 melanoma cells and of zebrafish embryo neural crest

mRNA expression analyses were performed in triplicates with SKMEL28 melanoma cells (3 experimental groups: untreated, Wnt3a pre-conditioned for 24 h, or 0.5 μM PKF115–584 (gifted by Novartis Oncology; Novartis Institutes for Biomedical Research, Cambridge, MA) pre-conditioned for 24 h) as described elsewhere [43, 44].

### siRNA transfections and lentiviral shRNA

Twenty nanometer siRNA was transfected into SKMEL28 melanoma cells using the riboxx-FECT (riboxx Life Sciences) reagent as recommended by the manufacturer. Briefly, 3 × 10^5^ melanoma cells were seeded per 6 well cavity, and the next day cells were transfected using combined Opti-MEM:siRNA and Opti-MEM:riboxx-FECT (12 μl per cavity) mixtures. The incubation time before addition to the cells was 15 min and the final volume was 2 ml. Cells were further incubated for 24 h before the usage in cellular assays. Lentiviral particles were generated and used as previously described for the shRNA mediated knockdown of β-catenin in melanoma cell lines [17].

### Scratch assay

5 × 10^5^ melanoma cells were seeded into 6well plates and grown until confluency followed by overnight serum starvation. Six hours before scratching the medium was exchanged with either a 1:1 mixture of Wnt3a-CM, 3T3-CM, or normal culture medium containing 0.5 μM PKF115–584 (Novartis Institutes for Biomedical Research, Cambridge, MA). Then a scratch was applied using a 200 μl standard pipette tip (Greiner Bio-One International, order No. 739290, Germany) and detached cells were removed by exchanging the medium once again with the corresponding treatment medium. Microphotographs were taken at 0, 12 and / or 24 h post scratching to measure the closed area using ImageJ (V 1.50b, NIH, USA). At least three biological replicates were measured in quadruplicates to analyze the closed area in % (mean +/− SD).

### Generation of cell aggregates, determination of primary aggregate formation

SKMEL28 cell suspensions (1 ml) were transferred into sterile gas permeable biofoil bags (Biofoil, Heraeus-Kulzer, Hanau, Germany) as described previously [13, 45]. Before sealing, either Wnt3a-conditioned medium or PKF115–584 (0.5 μM) was added into each bag. In the second set of experiments, the siRNA-transfected SKMEL28 cells were used without addition of drugs or conditioned medium. Bags were sealed and placed in open cylindrical plastic containers and were continuously kept in constant rotation (2 rpm) for 24 h using a roll mixer (Cellroll Integra Biosciences, Fernwald, Germany) in a cell incubator at 37 °C with 95% air and 5% CO_2_. To quantify the primary melanoma cell aggregation, aggregates were next transferred into six-well-plates and photographed with a microscope (Olympus IX50) mounted camera (Olympus E330). For morphometric analysis, 800–1200 aggregates per treatment were measured using AxioVision software (Zeiss, Oberkochen, Germany). Results were statistically evaluated using One way ANOVA, *p*-values of < 0.05 were considered as statistically significant.

### Primary melanoma cell migration

For determination of cell migration, the melanoma aggregates were placed into six-well-chambers in culture media (without de novo addition of Wnt3a or 0.5 μM PKF115–584) and further incubated for 24 h at 37 °C in a humidified incubator with 5% CO_2_. The aggregates and their homogeneous cellular outgrowths were photographed as described above. For morphometric analysis, the area of the remaining aggregate and the total area covering the aggregate plus the outgrown/migrated cells (representing the migrated cells) were determined in 200–300 aggregates per treatment group using AxioVision software (Zeiss). The capacity for migration (cell migration index) was calculated by dividing the area of the cellular outgrowth by the size of the aggregate and statistically evaluated (One way ANOVA) with multiple comparison (Dunn’s multiple testing).

### Luciferase reporter assay

For the assessment of Wnt/β-catenin signaling activity SKMEL28 melanoma cells were transfected with the Super8xTOPFlash plasmid as described previously [17, 46]. Cells were treated for 24 h before measuring the luciferase activity which was normalized to cell viability.

### Quantitative real-time PCR

RNA isolation from melanoma cells was performed using the NucleoSpin RNA Kit (Machery-Nagel, Dueren, Germany) followed by reverse transcription using Maxima reverse transcriptase (Thermo Fisher Scientific, Dreieich, Germany) as recommended by the manufacturers. Reverse transcribed cDNA was subjected to SYBR Green based real-time PCR as described previously [46] using the following primers: INHBA forward 5′- agctcagacagctcttaccaca-3″, INHBA reverse 5′ ttttccttctcctcttcagca-3′, CYR61 forward 5′-aaggagctgggattcgatg-3′. CYR61 reverse 5′- aggctccattccaaaaacag-3′. ANGPTL4 forward 5′- acgatggctcagtggacttc-3′. ANGPTL4 reverse 5′- cgtgatgctatgcaccttctc-3′, FABP7 forward 5′- aagtctgttgttagcctgga-3′, FABP7 reverse 5′- agggtcataaccattttgc-3′. CT values were normalized to 18 S rRNA expression.

### 3D spheroid assay

SKMEL28 melanoma cell aggregates were formed overnight before embedding the spheroids into a collagen matrix with or without 3T3 / 3T3-Wnt3a fibroblasts (1.5 × 10^5^ cells/ml) and cultured using RPMI with 10% FCS. Sprouting (invasion) of SKMEL28 cells was radially measured (*n* = 6) at 0 h and 48 h using ImageJ, and an invasion index calculated as ratio of the 48 h to the 0 h value.

### Organotypic skin reconstructs

Organotypic tissue skin reconstructs (TSR) were generated as described previously [40]. Briefly, human fibroblasts were seeded into a collagen type I (1.35 mg/ml) matrix before the addition of an epidermal layer consisting of HaCat keratinocytes and human melanoma cells. TSR were exposed to control medium (3T3-conditioned TSR medium) or Wnt3a-conditioned TSR culture medium for 14 days before fixation and parrafine embedding for immunohistochemical evaluation.

### Preparation of eggs, transplantation procedures, processing of the chick embryos

White leghorn chicken eggs were obtained from A. C. Weiss GmbH&Co. KG (Kirchberg/Iller, Germany). Egg handling and transplantations of SKMEL28 melanoma cells into the neural tube (untreated (*n* = 8), Wnt3a-preconditioned (*n* = 10), 0.5 μM PKF115–584-preconditioned (*n* = 12)), of BLM melanoma cells into the rhombencephalon (untreated (*n* = 8), 0.5 μM PKF115–584-pretreated (*n* = 8)), or of SKMEL28 melanoma cells into the rhombencephalon (control shRNA (*n* = 5), β-catenin shRNA (*n* = 5)) of stage 12/13 chick embryos according to Hamburger and Hamilton (HH) [47] were performed as previously described [15, 39]. For the shRNA experiment we used SKMEL28 cells with stable knockdown of β-catenin [17]. Chick embryos were further incubated after melanoma cell transplantation for 48 h (neural tube) or 96 h (rhombencephalon), respectively.

For the chorioallantoic membrane (CAM) metastasis assay eggs were incubated for 7 days at 38 °C with 60% humidity. On day 7 of chick embryo development a small window was made in the shell under aseptic conditions. The window was resealed with adhesive tape and eggs were returned to the incubator for 24 h. On day 8, the CAM was gently abraded with a sterile cotton swab to provide access to the mesenchyme and BLM melanoma cell suspensions (5 × 10^5^ cells) were seeded in 10 μl of PBS onto the upper CAM vasculature and further incubated at 37.5 °C for 7 days. On day 15 chick embryos were euthanized and CAM and livers removed for isolation of genomic DNA (gDNA). Melanoma cell derived human gDNA was detected by qPCR as described elsewhere [42].

### Immunohistochemistry and western blot

The following primary antibodies were used in this study: anti-HMB-45 (1:20, Dako/ Agilent, Hamburg, Germany), anti-β-catenin (#9562, 1:50, Cell Signaling Technology, Leiden, Netherlands) and anti-Ki67/MIB1 (1:100, Dako/ Agilent). Immunohistochemistry of a human melanoma tissue microarray (TMA) using an anti-β-catenin antibody (1:100, Cell Signaling #9562) was performed as described previously [46]. For western blotting the following antibodies were used: anti-Phospho-Akt (Ser473) (#4060), anti-Akt (#9272), anti-PTEN (#9188), anti-beta-actin (#3700) (all Cell Signaling Technology) and anti-beta-catenin sc-7963 (Santa Cruz).

All work with the TMA and human material was approved by the local ethics committee (305/2017BO2).

### Generation of Kaplan-Meier plots

Gene expression and clinical information were derived from The Cancer Genome Atlas (TCGA, skin melanoma dataset (*n* = 454)) (Network C.G.A., 2015). Patients were subclassified according to gene expression values (e.g. upper 11% and lower 11%). Five year survival rates were evaluated with the log-rank test. Multivariate Cox regression included gene expression, age, gender and pathologic stage. The survival curves were visualized by Kaplan-Meier plots.

### Statistical analyses

Statistical analyses were performed with GraphPad Prism 6.0 using the following tests: Fisher’s exact test, One way ANOVA, Mann-Whitney. *p*-values < 0.05 were considered as statistically significant.

## Results

### β-catenin is prominently expressed in primary human melanomas at the invasive front

**Fig. 1:**
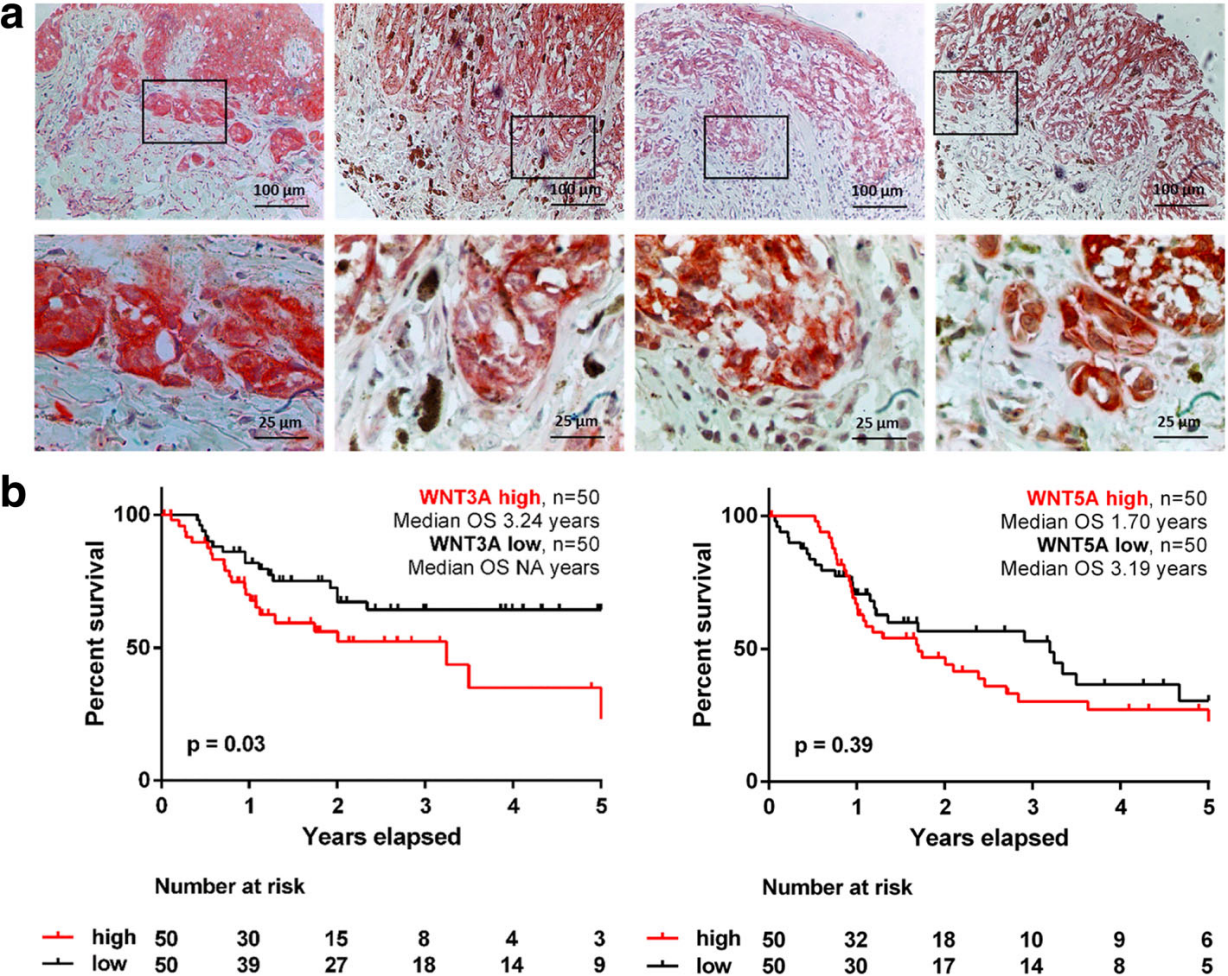
β-catenin is expressed in primary human melanomas in particular in cells of the invasive front. **a** Cytoplasmic expression of β-catenin in primary melanomas. A stronger expression of β-catenin in melanoma cells of the invasive front was observed by immunohistochemistry when compared to the bulk cells of the primary melanoma. Melanoma cells of the invasive front with a high expression of β-catenin had a spindle-like, mesenchymal morphology. Overview (upper row) and higher magnification of the invasive front (lower row) of four representative primary melanomas. **b** Overall survival (OS) analysis of the TCGA data set for the top and bottom (*n* = 50 each) WNT3A and WNT5A expressing melanomas reveal a significantly worse OS for WNT3A-high (*p* = 0.03) but not WNT5A-high melanoma patients (*p* = 0.39)

In our previous study, we demonstrated cytoplasmic expression of β-catenin in human primary melanomas using a melanoma tissue microarray [46]. We re-evaluated the expression of β-catenin at the invasive front in the primary melanomas. We could frequently detect a strong expression of β-catenin in melanoma cells at the invasive front (Fig. 1a). Interestingly, invasive melanoma cells depicting a high expression of β-catenin had a rather spindle-like, mesenchymal morphology. This observation led us to hypothesize that Wnt ligands and Wnt-signaling could be actively involved in melanoma cell invasion. Interestingly, analysis of the TCGA melanoma data revealed a significant (*p* = 0.03) inverse association of WNT3A gene expression with the five-year overall survival (OS) of melanoma patients when comparing the specimens with the highest (top 11%, *n* = 50) with lowest (bottom 11%, *n* = 50) gene expression (Fig. 1b). In contrast, WNT5A gene expression did not reach significance although the WNT5A^high^ group showed by trend a reduced median OS of 1.70 years (*p* = 0.39). Further stratification of the WNT3A^high^ and WNT3A^low^ melanoma tumors by their PTEN expression levels yielded a reduced five-year overall survival (OS) for patients with high WNT3A and low PTEN expression levels. The same was found for WNT3 sharing 84% amino acid identy with WNT3A. No significant difference in OS by PTEN stratification was observed for WNT3A^low^ melanomas demonstrating an important interaction of PTEN and the activated Wnt signaling pathway in melanoma (Additional file 1: Figure S1).

### The agonist Wnt3a enhances, while the β-catenin inhibitor PKF115–584 blocks neural crest migration of melanoma cells in the chick embryo in vivo

**Fig. 2:**
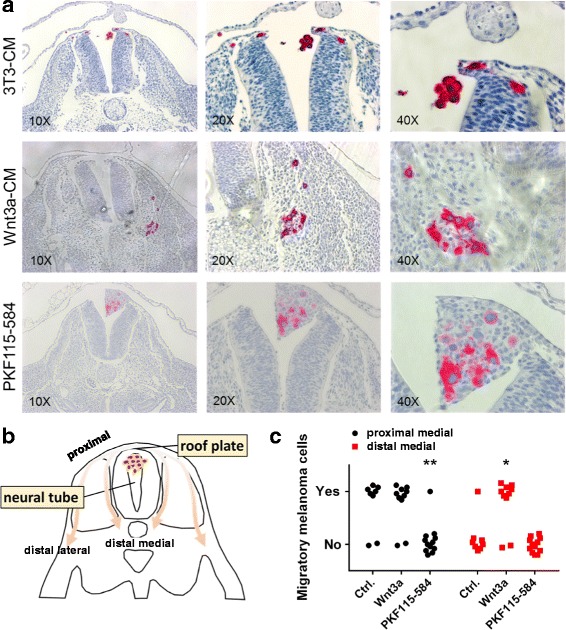
Wnt3a enhances and PKF115–584 blocks neural crest migration of SKMEL28 melanoma cells in the chick embryo in vivo. **a** Control treated (*n* = 8), Wnt3a (*n* = 10) or 0.5 μM PKF115–584 (*n* = 12) pre-conditioned SKMEL28 melanoma cell aggregates were injected into the neural tube of stage 12/13 HH chick embryos and further incubated for 48 h. Embryos were formalin-fixed and paraffine-embedded (FFPE) before sectioning and staining for HMB45 (red) to identify the SKMEL28 melanoma cells. In 6/8 embryos, untreated HMB45-positive SKMEL28 cells integrated into the roof plate of the neural tube and migrated mainly along the proximal medial neural crest cell pathway. Wnt3a pre-conditioned SKMEL28 cells similarly integrated into the roof plate and predominantly performed medial neural crest migration in 8/10 embryos (not significant, Fisher’s Exact Test, compared to the untreated cells). An increased number of HMB45-positive Wnt3a pre-conditioned cells were detected along the distal medial neural crest cell pathway close to the notochord and the developing para-aortic sympathetic ganglia. 0.5 μM PFK115–584 pre-conditioned, HMB45-positive SKMEL28 cells formed compact tumor nodules in the dorsal lumen of the neural tube and were attached to and integrated into the roof plate and the adjacent mesenchyme between the roof plate and the surface ectoderm. Only in 1/12 embryos single emigrating cells were detected along the proximal medial neural crest cell pathway in direct vicinity to the lateral roof plate (*p* < 0.01, Fisher’s Exact Test, compared to the untreated cells). **b** Scheme showing the neural crest-like migration patterns of melanoma cells out of the roof plate after injection into the neural tube. **c** Quantification of chick embryos showing SKMEl28 melanoma cells that migrated to proximal or distal sites of the medial migration pathway after different pre-treatments (asterisks define *p*-values calculated by Fisher’s exact test)

To analyze whether Wnt signaling was involved in neural crest cell migration of melanoma cells, we generated aggregates from a cell suspension of SKMEL28 melanoma cells kept in gas-permeable biofoil bags in double rotation for 24 h. During aggregation, the cells were cultured in control medium (3T3 conditioned) or stimulated with conditioned medium of Wnt3a (agonist) secreting 3T3 fiboblasts (Wnt3a-CM) or PKF115–584 (antagonist) to the medium. After 24 h, the aggregates were transplanted into the embryonic neural tube of stage 12/13 HH chick embryos. As shown previously [8], 48 h after transplantation control treated SKMEL28 cells (immunohistochemically identified in the chick embryos by anti-HMB45 staining) spontaneously integrated into the roof plate of the neural tube in 6/8 transplanted embryos and were detected mainly at proximal sites along the medial neural crest migratory pathway (Fig. 2a-c). A similar migration pattern was observed in the Wnt3a-CM pre-conditioned SKMEL28 cells: integration into the roof plate and predominantly medial neural crest migration in 8/10 transplanted embryos (*p* = 1.0, Fisher’s Exact Test, compared to the control treated cells; Fig. 2a-c). However, we detected an increased number of single and clustered SKMEL28 melanoma cells along distal sites of the medial neural crest cell pathway close to the notochord and the developing para-aortic sympathetic ganglia upon Wnt3a stimulation (*p* = 0.0152, Fisher’s Exact Test). This is in line with the previously described enhanced neural crest migration of B16-F1 melanoma cells upon stimulation with BMP-2 [13]. PFK115–584 pre-conditioned SKMEL28 cells showed a completely different result: 48 h after transplantation into the neural tube the cells had formed compact tumor nodules in the dorsal lumen of the neural tube and were attached to and integrated into the roof plate and the adjacent mesenchyme between the roof plate and the surface ectoderm (Fig. 2a-c). However, only in 1/12 of transplanted embryos we detected few single emigrating cells along the proximal medial neural crest cell pathway in direct vicinity to the lateral roof plate (*p* = 0.0044, Fisher’s Exact Test, compared to the untreated cells). To confirm the viability of the melanoma cells, we performed anti-Ki67 (MIB1) immunohistochemistry. Almost all melanoma cells in the chick embryo were MIB1-positive, clearly demonstrating the proliferative activity and viability of the transplanted cells (Additional file 1: Figure S2A). Immunohistochemical staining with anti-β-catenin showed that the PKF115–584 pre-treatment caused a reduced cytoplasmic expression of β-catenin in the HMB45 positive melanoma cells (Additional file 1: Figure S2B).

### Wnt3a increases and PKF115–584 decreases melanoma cell migration

**Fig. 3:**
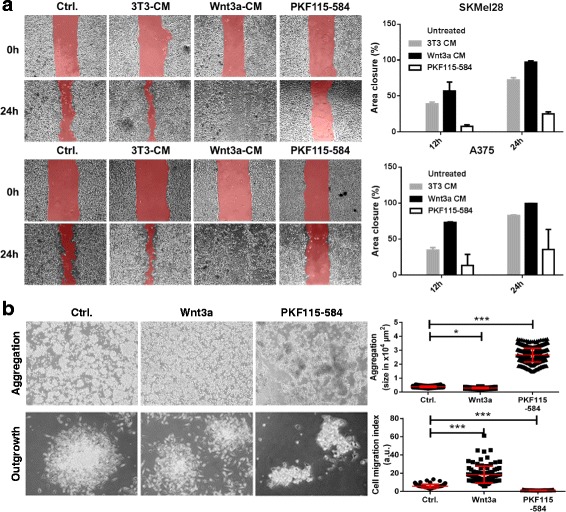
Wnt3a induces and PKF115–584 inhibits migration and typical features of EMT of melanoma cells in vitro. **a** Scratch assay of human SKMEL28 (upper row) and A375 (lower row) melanoma cells showing increased migration upon stimulation with Wnt3a conditioned medium (Wnt3a CM) and decreased migration upon treatment with PKF115–584) when compared to untreated and 3T3-medium conditioned cells (3T3-CM). **b** Upper row/ Aggregation: Aggregates were generated from SKMEL28 cells (untreated, or stimulated by Wnt3a or 0.5 μM PKF115–584 during aggregation), photographed and measured after 24 h. Wnt3a decreased and PKF115–585 increased the aggregate size (*: *p* < 0.05; ***: *p* < 0.001, One way ANOVA). Depicted: aggregate size in μm^2^±SD. Lower row/ Outgrowth: The SKMEL28 aggregates were re-incubated for 24 h in 6-well-plates and photographed. Around each aggregate a single layered outgrowth of emigrating cells had formed. The areas of aggregates and cellular outgrowths were measured and the migration capacity (cell migration index) determined by dividing the area of the outgrowth by the area of the aggregate. Wnt3a increased and PKF115–584 decreased the cell migration index (***: *p* < 0.001, One way ANOVA). Depicted: cell migration index±SD

To analyze the impact of Wnt stimulation and inhibition on melanoma cell migration, we conducted wound closure experiments (scratch assays) with serum-starved human SKMEL28 (upper row) and A375 (lower row) melanoma cells (Fig. 3a). The cells were either left untreated (untreated control), pre-conditioned with 3T3-medium (3T3-CM), Wnt3a-3T3 medium (Wnt3a-CM), or for 4 h with 0.5 μM PKF115–584. The wound was photographed at 0 h, 12 h and at 24 h, and the closed area (migrated cells) was morphometrically measured. After 24 h, we observed significantly increased migration upon stimulation with Wnt3a-CM, and a significantly decreased migration upon treatment with PKF115–584. To that effect there is a clear impact of fibroblast derived Wnt3a on melanoma cell migration. A 4 h treatment with 0.5 μM PKF115–584 did not dramatically reduce melanoma cell viability within 24 h after scratching (Additional file 1: Figure S3). However, massive cell death was observed when the incubation was prolonged, confirming our former studies [17]. A third cell line SKMEL19 showed the same trend, however the increased wound closure upon Wnt3a-CM treatment was not significant (Additional file 1: Figure S3A). Scratch assay using co-cultured fluorescence-labeled 3T3 and 3T3-Wnt3a fibroblast cells with 451Lu, BLM or A375 melanoma cells confirmed our findings (Additional file 1: Figure S3B). Interestingly, also the Wnt3a producing 3T3 cells themselves exhibited an increased migratory potential into the gap of the wound compared to the 3T3 control cells.

### Wnt3a decreases and PKF115–584 increases aggregation of melanoma cells

During neural crest induction EMT is characterized by two important morphological steps: (i) delamination or decrease of adhesion (ii) induction of migration. To analyze these two steps (adhesion and migration) in more detail in a more physiological 3D–environment, we performed two sets of experiments in vitro. In the first experiment, we determined whether Wnt-signaling influenced melanoma cell aggregation. To this end we generated aggregates as described above. The cells were either left untreated or stimulated by the addition of Wnt3a-CM (agonist) or PKF115–584 (antagonist) to the medium. After 24 h, the spontaneously generated aggregates were photographed and measured (Fig. 3b, upper row). The control SKMEL28 cells homogenously formed aggregates (*n* = 962 measured aggregates, 3863 ± 646 μm^2^). We found a significantly decreased aggregate size by 26% after exposure of the SKMEL28 cells to the agonist Wnt3a (*n* = 1199 measured aggregates, 2823 ± 485 μm^2^) and a significantly increased aggregate size after exposure to the antagonist PKF115–585 (*n* = 792 measured aggregates, 26,537 ± 4927 μm^2^).

### Wnt3a increases and PKF115–584 decreases primary melanoma cell migration in vitro

To test the second step of EMT (induction of migration), we performed a cell outgrowth assay comparable to the original description of embryonic EMT in vitro [6]. SKMEL28 aggregates generated after 24 h of aggregation time as above (either untreated, conditioned with Wnt3a-CM or pre-treated with PKF115–584) were seeded into 6-well-plates, incubated for another 24 h (in new medium without further stimulation, with either Wnt3a or PKF115–584), and photographed. Around the Wnt3a preconditioned and untreated SKMEL28 aggregates a pronounced coherent, single layered outgrowth of emigrating cells had formed, which was less prominent in the PKF115–584 treated group (Fig. 3b, lower row). For quantitative evaluation, the areas of aggregates and respective outgrowths were measured by morphometry. Wnt3a significantly increased the cell migration index (*n* = 100 aggregates measured, 18.42 ± 9.23 [a.u.]) and PKF115–584 significantly decreased (*n* = 313 aggregates measured, 1.38 ± 0.2 [a.u.]) the cell migration index of SKMEL28 aggregates when compared to the control group (*n* = 90 aggregates measured, 5.80 ± 2.01 [a.u.]).

### Wnt3a enhances melanoma cell invasion in organotypic skin reconstructs

**Fig. 4:**
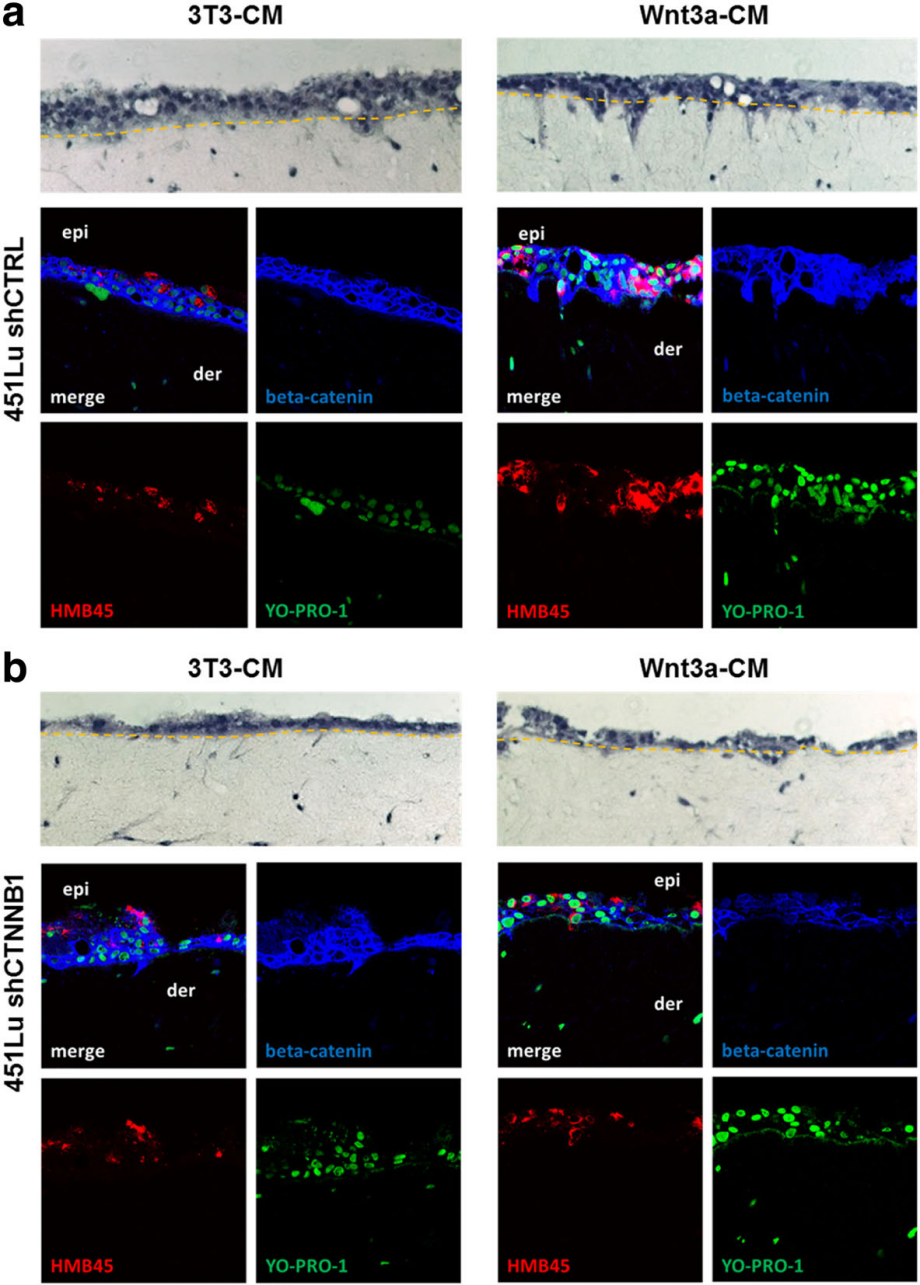
Wnt3a induces invasive growth of melanoma cells in organotypic tissue skin reconstructs (TSR). **a** 451 LU melanoma cells were seeded together with HaCat epidermal cells onto a layer of collagen I embedded human fibroblasts. TSR exposed to Wnt3a conditioned medium (Wnt3a-CM) showed a pronounced invasive morphology in the H&E staining (upper pictures) when compared to cells exposed to control medium (3T3-CM). Immunofluorescence stainings for HMB45 (red) and beta-catenin (blue) identified melanoma cells (HMB45+), revealed beta-catenin expression levels and verified the invasion of single 451 LU cells from the epidermis (epi) into the dermal part (der). Nuclei were stained with YO-PRO-1 (green). **b** Knockdown of beta-catenin (blue) with shRNA (shCTNNB1) reduced the invasion of 451 LU melanoma cells (HMB45+, red) into the dermal part of the TSR

Similarly, melanoma cells that were seeded together with HaCat keratinocytes onto the dermal part of organotypic tissue skin reconstructs (TSR) showed increased invasion of melanoma cells into the dermis when the medium was pre-conditioned by Wnt3a-producing fibroblasts (3T 3 cells) in comparison to the conditioning with control 3T3 cells. This effect was seen using BLM, A375 and 451Lu melanoma cells (Fig. 4a and Additional file 1: Figure S2). Knockdown of β-catenin using a lentiviral shRNA vector abolished the invasive capacity of the melanoma cells independent of the preconditioning of the medium (Fig. 4b and Additional file 1: Figure S2). Additional immunoflourescence stainings of TSR with HMB45-positive 451Lu cells revealed that melanoma cells mostly resided in the epidermal layer with keratinocytes when TSR were treated with 3T3-conditioned medium or after knockdown of β-catenin. In contrast, Wnt3a-conditioned medium induced a more invasive phenotype of the 451Lu cells including invasion into the dermal (collagen type I) matrix. Staining for β-catenin showed the typical membranous staining in the HaCat epidermal layer. After the knockdown by lentiviral expression of CTNNB1 specific shRNA in the 451Lu melanoma cells a reduced cytoplasmic and nuclear protein level became evident when compared with invading 451Lu melanoma cells that showed a higher expression of cytoplasmic and nuclear β-catenin (Fig. 4a-b).

### A common set of genes is expressed during neural crest induction in the zebrafish embryo and upon Wnt induction in SKMEL28 melanoma cells

To further elucidate whether a common set of genes was expressed during neural crest induction in the zebrafish embryo and in human melanoma cells upon Wnt3a stimulation, we conducted mRNA expression analyses of human SKMEL28 melanoma cells (untreated, Wnt3a, or PKF115–584 pre-conditioned) and of isolated zebrafish embryo neural crest. Forty four overlapping genes were unanimously differentially expressed in SKMEL28 after treatment with Wnt3a or Wnt−/β-catenin inhibition by PKF115–584 (Additional file 2: Table S1 and Additional file 1: Figure S5A). By comparing these 44 genes to the gene expression pattern of the zebrafish neural crest, we found an overlap of the following six genes: GTP binding protein over-expressed in skeletal muscle (GEM), angiopoietin-like 4 (ANGPTL4), dual specificity phosphatase 5 (DUSP5), pleckstrin homology-like domain, family A, member 2 (PHLDA2), interferon-induced protein 44 (IFI44), and fatty acid binding protein 7 (FABP7).

A comparison of the 44 differently expressed genes with the gene expression patterns of distinct melanoma cell phenotypes as determined by Hoek et al. (invasive or proliferative, [48]) revealed an overlap of three genes (cysteine-rich, angiogenic inducer, 61 (CYR61), angiopoietin-like 4 (ANGPTL4), and inhibin beta A (INHBA)). All three were significantly upregulated in cells with an invasive phenotype (Additional file 1: Figure S5B). Out of these eight candidate genes we chose four candidates with a possible impact on EMT in the melanoma cells for further investigation: INHBA, FABP7, ANGPTL4, and CYR61. The increased expression in three of the genes in the invasive cell lines compared to the proliferative cell lines is in contradiction with the up-regulation after Wnt3a-CM stimulation in SKMEL28 cells and our observed pro-migratory effects. It is important to know that the phenotypes used by Hoek et al. were based on different model systems compared to our experiments. However, they revealed genes that are involved in the biology of melanoma cells.

**Fig. 5:**
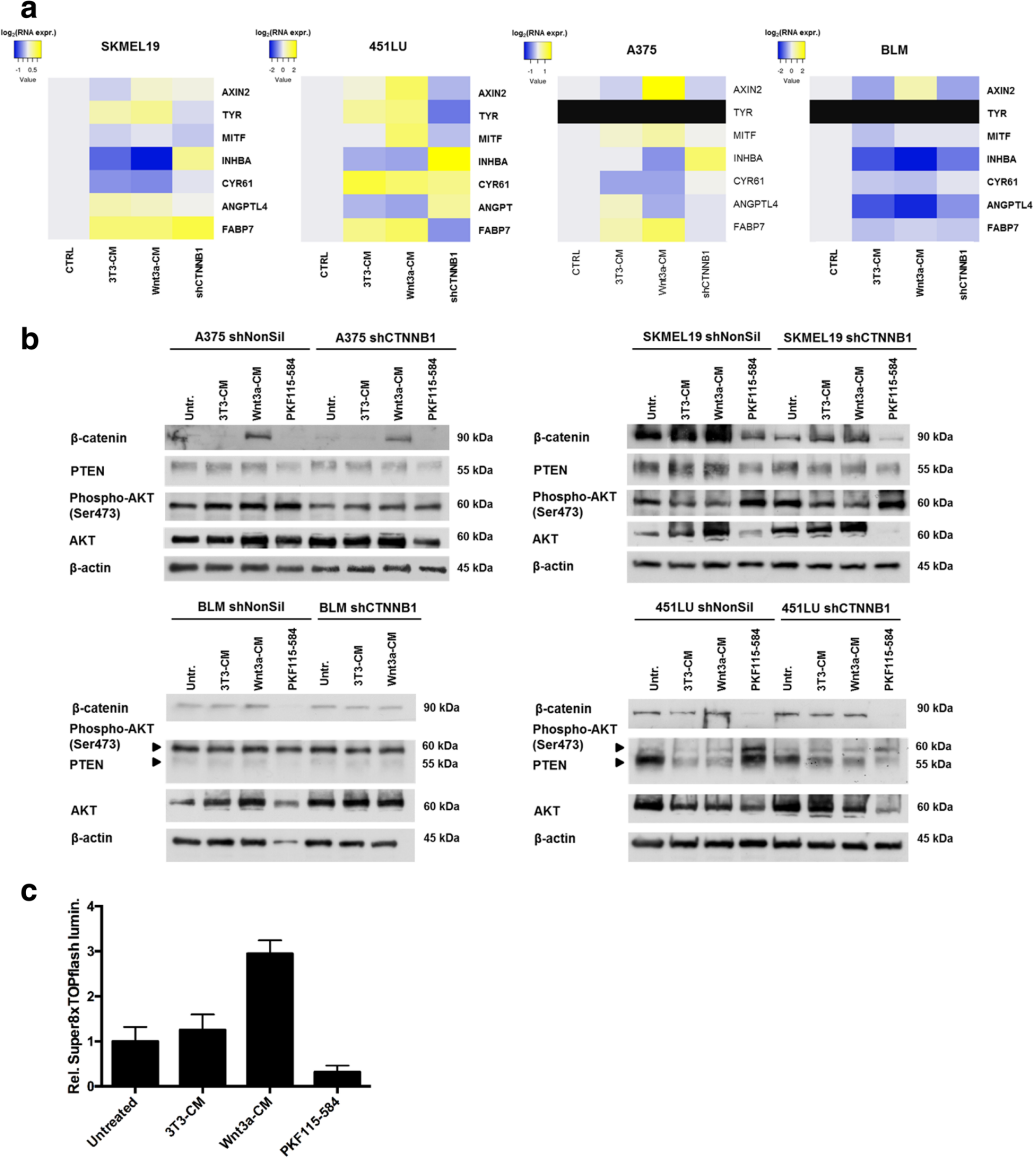
Gene expression during neural crest induction in the zebrafish embryo and upon Wnt stimulation in SKMEL28 melanoma cells, and clinical significance. **a** Heatmaps of real-time qPCR gene expression analysis of the melanoma cell lines SKMEL19, 451 LU, BLM and A375 for AXIN2. TYR, MITF and the four genes INHBA, CYR61, ANGTPL4 and FABP7. Log2 transformed x-fold expression values were used for color-coding. Yellow: upregulated gene expression; blue: downregulated gene expression at 24 h treatment of the melanoma cells; black: not detectable **b** Western blot analysis to detect protein levels of beta-catenin, PTEN, phospo-Ser473 (AKT) and AKT in the melanoma cell lines A375, SKMEL19, BLM and 451 LU after treatment with 3T3-CM, 3T3-Wnt3a, or 0.5 μM PKF115–584 for 24 h. Beta-actin was used as loading control. Small-hairpin knockdown cells served as samples to investigate the dependence on beta-catenin. **c** Luciferase reporter assay (Super8xTOPFlash) indicates a 3-fold activation of the canonical Wnt−/β-catenin signaling pathway after stimulation of SKMEL28 cells with Wnt3a-conditioned medium (3T3-Wnt3a). PKF115–584 treatment (0.5 μM for 12 h) significantly inhibited reporter activity. **: *p* < 0.01, ***: *p* < 0.001, One way ANOVA

To test the functionality of our cell treatments we used a firefly luciferase reporter assay (Super8xTOPFlash) which measures the transcriptional activity of canonical Wnt signaling. As expected, treatment of the SKMEL28 melanoma cells with Wnt3a-conditioned medium but not with 3T3-conditioned medium induced the transcriptional activity of the Wnt/β-catenin signaling pathway (Fig. 5c). Moreover, treatment with 0.5 μM PKF115–584 for 12 h more than halved the luminescence signal in the reporter assay indicating the inhibitory effect of PKF115–584 on the Wnt/β-catenin signaling pathway. Another method to measure the activity of the Wnt/β-catenin signaling pathway is the quantitative RNA expression analysis of the target gene AXIN2 by real-time qPCR. Treatment of four different melanoma cell lines with Wnt3a-CM induced AXIN2 expression in comparison with 3T3-CM treated melanoma cells (Fig. 5a). Vice versa knockdown of β-catenin reduced in three out of four cell lines the expression of AXIN2, again confirming the interfering of our treatments with the Wnt/β-catenin signaling pathway. MITF expression was only slightly induced by Wnt3a-CM and an increased expression of TYR could only be detected in SKMel19 and 451 LU cells. TYR was not detectable in A375 and BLM melanoma cells. The down-regulation of INHBA, CYR61 and ANGPTL4, which was found in SKMEL28 melanoma cells after treatment with Wnt3a-CM could be confirmed in these four cell lines by real-time qPCR when compared to the 3T3-CM samples. FABP7 was found to be up-regulated, again supporting the results obtained from SKMEL28 melanoma cells (Fig. 5b). On protein level the treatment of melanoma cells with Wnt3a-CM increased β-catenin expression whereas PKF115–584 treatment reduced β-catenin as expected. Interestingly, all melanoma cell lines and all conditions showed an activation of the PI3K signaling pathway as indicated by phosphorylated Ser473 of AKT although only BLM did not express PTEN in clearly detectable amounts (BLM is known to have a methylated PTEN promoter). PKF115–584 induced further AKT phosphorylation which hints at a putative counter regulation to the reduced β-catenin levels, since GSK3 is a target of AKT and Ser9 phosphorlyation of GSK3 stabilizes β-catenin.

Since we observed a close correlation between the Wnt3a-CM or PKF115–584 treatment and the expression of pigmentation related genes (DCT, TYR, TYRP1, MITF) in our SKMEL28 microarray data, we analyzed the TCGA melanoma RNAseq expression data concerning MLANA expression. We chose the top and bottom 11% (*n* = 50) of melanomas in terms of MLANA expression and identified the differentially expressed genes considering our 44 differentially expressed genes. We found 22/44 of our identified genes (including INHBA, CYR61 and ANGPTL4) to be significantly co-expressed (Additional file 2: Table S1). This supports the biological in vivo relevance of our investigated gene pattern in melanoma.

### siRNA knock-down confirms the double role of genes simultaneously involved in embryonic neural crest induction and melanoma cell EMT

**Fig. 6:**
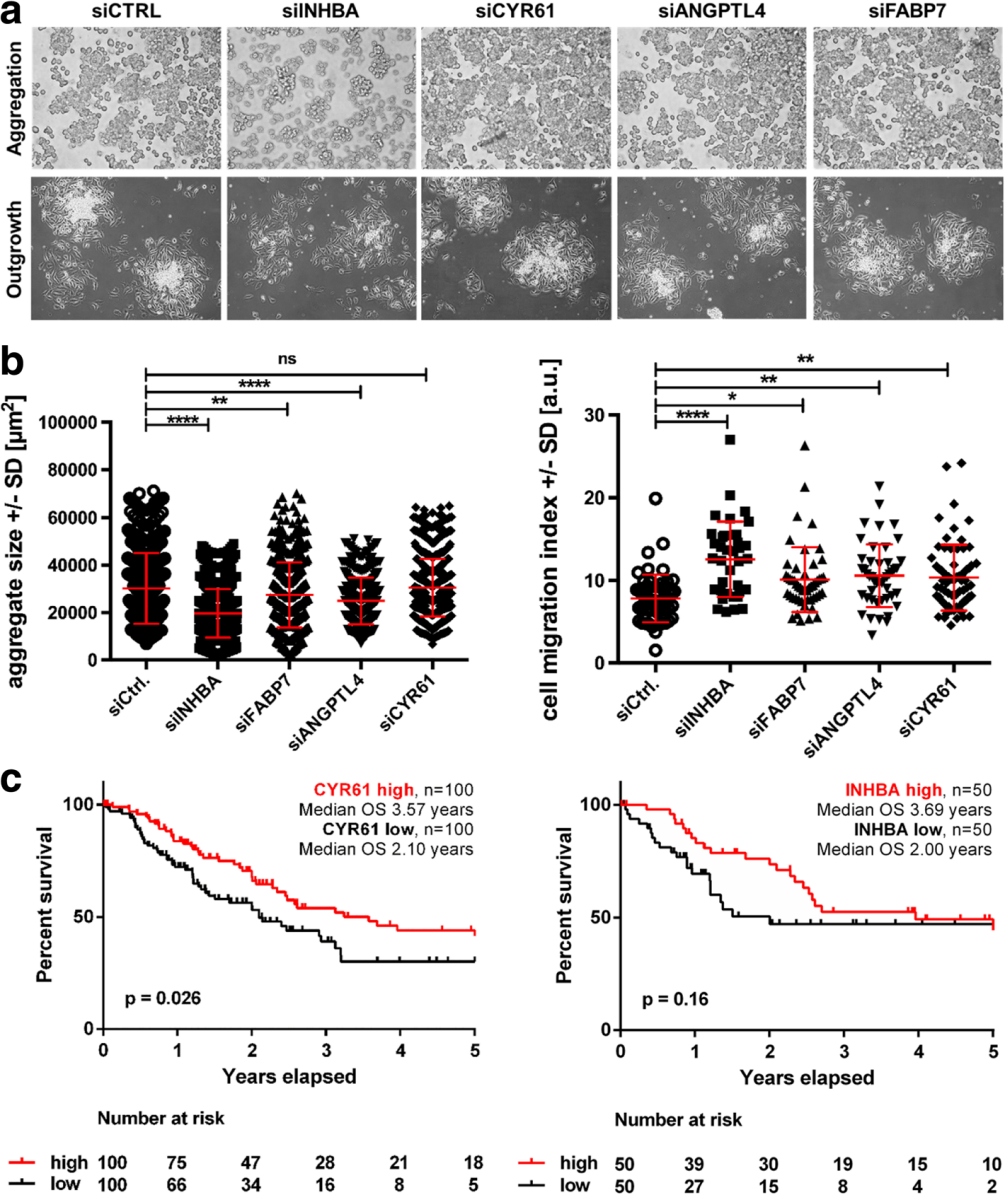
siRNA knock-down confirms the double role of genes simultaneously involved in embryonic neural crest induction and melanoma cell adhesion. **a** SKMEL28 cells aggregated during 24 h of roller culture after knockdown of the four gene candidates (upper row) and were microphotograped (10X magnification) Further cultivation of the aggregates for 24 h was used to detect the outgrowth of migratory cells (10X magnification). **b** For all four knocked-down genes a significant decrease of aggregate size (left diagram) compared to the siCtrl SKMEL28 cells was observed (ns: not significant, **: *p* < 0.01, ****: *p* < 0.0001, One way ANOVA). Depicted in red: mean aggregate size in μm^2^±SD. An increased cell migration index (right diagram) was detected for all four knocked-down genes in the transfected SKMEL28 cells compared to siCtrl SKMEL28 cells (*: *p* < 0.05, **: *p* < 0.01, ****: *p *< 0.0001, One way ANOVA). Depicted in red: mean cell migration index±SD. **c** Kaplan-Meier plots of CYR61- or INHBA-expression in primary melanomas demonstrating a correlation of gene expression with overall survival of melanoma-afflicted patients

In a next step, we down-regulated the four selected genes above (INHBA, CYR61, ANGPTL4, andFABP7) using siRNA in SKMEL28 cells to analyze their possible influence on melanoma cell adhesion and migration. We observed a significant decrease of aggregate size for the knockdown of INHBA, ANGTPL4 and to a lesser extent for FABP7 (siCtrl: *n* = 440 measured aggregates, 30,206 ± 14,892 μm^2^; siINHBA: *n* = 643 measured aggregates, 19,668 ± 10,191 μm^2^; siFABP7: *n* = 516 measured aggregates, 27,421 ± 13,718 μm^2^; siANGPTL4: *n* = 356 measured aggregates, 24,921 ± 9825 μm^2^; siCYR61: *n* = 720 measured aggregates, 30,531 ± 12,064 μm^2^; one-way ANOVA, Fig. 6a, b). These results suggest a role in melanoma cell adhesion for INHBA, and ANGPTL4. More important and mostly in line with our previous data (but opposing the data of Hoek et al. (Fig. 3b, Additional file 1: Figure S5B)), we detected a significantly increased outgrowth and thereby migration capacity for all four different knockdown conditions, especially with siRNA specific for INHBA, ANGPTL4 and CYR61 (siCtrl: *n* = 56 aggregates measured, 7.82 ± 2.88 [a.u.]; siINHBA: *n* = 36 aggregates measured, 12.56 ± 4.58 [a.u.]; siFABP7: *n* = 50 aggregates measured, 10.10 ± 3.93 [a.u.]; siANGPTL4: *n* = 46 aggregates measured, 10.58 ± 3.82 [a.u.]; siCYR61: *n* = 69 aggregates measured, 10.35 ± 4.0 [a.u.], Fig. 6a and b). This demonstrates a regulatory function for INHBA, FABP7, ANGPTL4, and CYR61 in melanoma cell migration. The least effect was again detected for the knockdown of FABP7. Since we also measured increased migratory behavior after knockdown of FABP7 (although this gene was upregulated with Wnt3a-CM treatment) a composite of gene expression alterations after Wnt3a-CM might be responsible for the effect of increased migration and reduced homotypic cell adhesion.

Next, we investigated a possible impact of the four selected genes (INHBA, FABP7, ANGPTL4, and CYR61) on overall survival of melanoma patients [49]. For this purpose, we analyzed melanoma gene expression levels and clinical information from The Cancer Genome Atlas (TCGA, skin melanoma dataset (*n* = 454)) [50]. The survival curves of CYR61 and INHBA were visualized by Kaplan-Meier plots (Fig. 6c). Indeed, a high expression (upper and lower 22%, *n* = 100 per group) of CYR61 in primary melanomas was significantly associated with increased 5-year overall survival in the corresponding patients (median OS of 3.57 versus 2.10 years). We detected a strong trend for increased overall survival for patients with primary melanomas bearing a high expression level (median OS of 3.67 versus 2.0 years; *n* = 50 per group) of INHBA (Fig. 6c). No differences in OS were found for ANGPTL4 and FABP7 stratifications (data not shown).

### β-catenin is expressed in the cytoplasm of malignantly invading melanoma cells during brain metastasis in vivo in the chick embryo; the β-catenin antagonist PKF115–584 potently inhibits metastasis

**Fig. 7:**
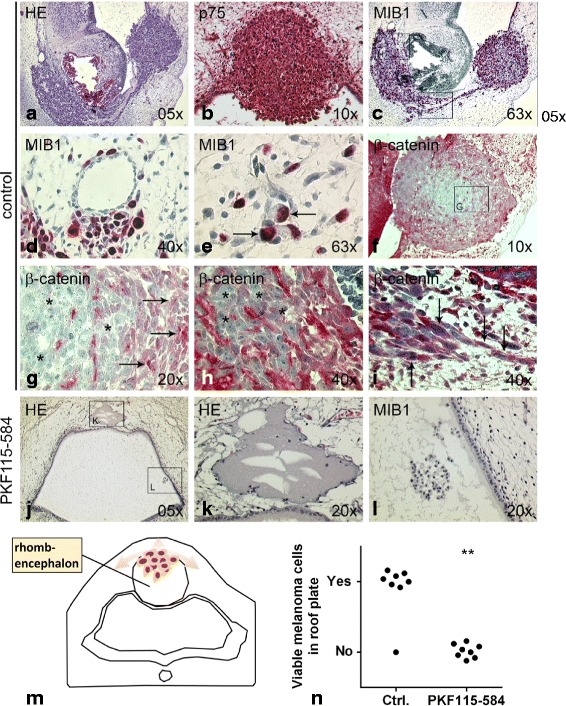
Expression of β-catenin in BLM melanoma cells during brain metastasis in vivo*.* Untreated (*n* = 8) or 0.5 μM PKF115–584 pre-conditioned (*n* = 8) human BLM melanoma cell aggregates were injected into the IV^th^ ventricle (rhombencephalon) of the stage 12/13 HH chick embryo (**m**). After 96 h of further incubation the embryos were analyzed for tumor formation and melanoma cell invasion. **a** Untreated BLM cells had formed small tumor nodules in the roof plate of the rhombencephalon and infiltrated the neighboring host tissues in 7/8 chick embryos. **b** The HMB45-negative BLM cells the neural crest marker p75 (red cytoplasmic staining). **c-e**: BLM cells were almost 100% MIB1-positive (red nuclear staining) and were detected close to (**d**) or within (**e**, arrows pointing at BLM cells) blood vessels. **f-i** Immunohistochemistry for anti-β-catenin (**f**, red staining) showed that in the central tumor nodule as well as the metastatic nodule in the region of the floor plate the MIB1-positive BLM cells with a compact, epithelial-like morphology (asterisks in **g** and **h**) had only a weak cytoplasmic expression of β-catenin, while the cells in the outskirt of the tumor nodule and cells invading the host tissues in the mesenchyme along the neuroepithelium with a stretched, mesenchymal-like morphology (arrows in **g** and **i**) showed a prominent β-catenin expression. **j-l** In 8/8 embryos injected with PKF115–584 pre-conditioned BLM cells only an empty space as reminder of the former tumor nodule was detected after 96 h (**j, k**). Only few PKF115–584 pre-conditioned, MIB1-negative BLM cells and aggregates with morphological signs of apoptosis could be distinguished at this point in the lumen of the IVth ventricle floating among cellular debris of dead BLM cells (**l**). **m** Scheme of the injection of melanoma cells into the rhombencephalon of chick embryos. **n** Qualitative enumeration of chick embryos showing viable BLM melanoma cells in the roof plate with invading melanoma cells as shown in A-L. (asterisks define *p*-values calculated by Fisher’s exact test)

Next, we sought to determine if the inhibition of the Wnt-signaling pathway with PKF115–584 also had an impact on tumor formation, malignant invasion of melanoma cells and metastasis in vivo. We transplanted untreated (*n* = 8) or PKF115–584 pre-conditioned (*n* = 8) human BLM melanoma cell aggregates generated as described above into the IV^th^ ventricle (rhombencephalon) of the stage 12/13 HH chick embryo, and analyzed the embryos for possible tumor formation and invasion after an additional 96 h of incubation. Analogous to former results [39], the untreated BLM cells formed small tumor nodules in the roof plate of the rhombencephalon and infiltrated the neighboring host tissues (Fig. 7a). Since BLM cells are HMB45-negative the neural crest marker p^75^ was used for melanoma cell identification (Fig. 7b). Close to 100% of the BLM cells were MIB1-positive (Fig. 7c). We detected a high number of invading BLM cells in close vicinity to blood vessels (Fig. 7d) and some BLM cells had even entered the vasculature and thus actively metastasized by transendothelial migration (Fig. 7e). This is in line with the reported melanoma cell angiotropism during metastasis [51] and the role of β-catenin in transendothelial migration [52]. To determine the role for β-catenin during tumor formation and invasion of BLM cells in the chick brain, we stained serial sections for β-catenin. Immunohistochemistry revealed that in the tumor nodule the MIB1-positive, proliferating, centrally located BLM cells depicting a compact, epithelial-like morphology (Fig. 7c) had only a weak cytoplasmic expression of β-catenin (Fig. 7f, parallel slide of Fig. 7c), while the cells in the outskirt of the tumor nodule with a spindle-like, invasive morphology showed a more prominent cytoplasmic β-catenin expression (Fig. 7g, compare to Fig. 1). The same phenomenon could be observed in the region of the floor plate of the rhombencephalon, where invading BLM cells had already formed a secondary tumor nodule/metastasis. Here too, compact, epithelial-like cells had only a weak, and stretched, mesenchymal-like cells a more prominent cytoplasmic β-catenin expression (Fig. 7h). Single BLM cells with a similar stretched, mesenchymal morphology invading the host tissues in the mesenchyme along the neuroepithelium again showed a prominent expression of β-catenin (Fig. 7i, compare Fig. 1).

Further, we observed that in all eight transplanted embryos the BLM cells pre-treated with the β-catenin inhibitor PKF115–584 had obviously formed a tumor nodule at the dorsal midline of the roof plate in the IV^th^ ventricle (as expected), but had subsequently mostly died, leaving only an empty space as reminder of the former tumor nodule (Fig. 7j, k). The few remaining PKF115–584 pre-conditioned cells were floating among cellular debris of dead BLM cells in the lumen of the IV^th^ ventricle and showed no signs of proliferation (negative for Ki67/MIB1, Fig. 7l).

**Fig. 8:**
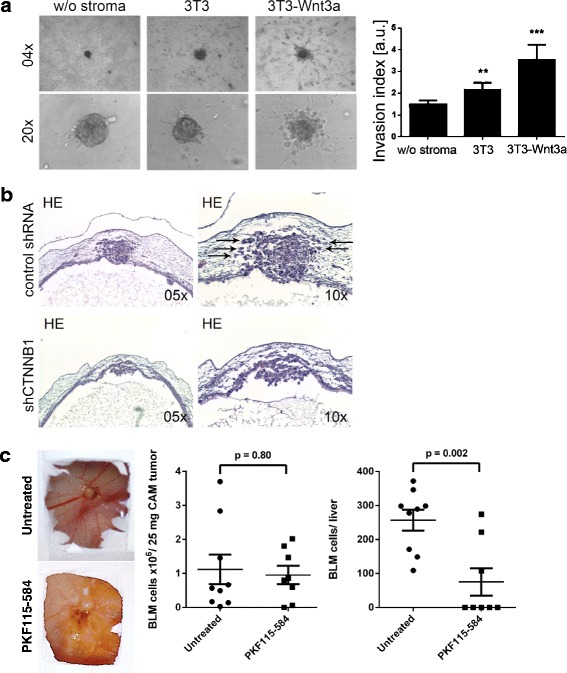
Knock-down of β-catenin with shRNA in SKMEL28 cells inhibits brain metastasis formation in the rhombencephalon of the chick embryo. **a** SKMEL28 aggregates were embedded into a collagen matrix with or without 3T3 or 3T3-Wnt3a stroma cells. Sprouting of SKMEL28 cells was measured at 0 h and 48 h, and an invasion index calculated as ratio of the 48 h to the 0 h value. 3T3 stroma cells, and more prominently, 3T3-Wnt3a stroma cells increased sprouting of SKMEL28 cells (**: *p* < 0.01; ***: *p* < 0.001, One way ANOVA). **b** Control shRNA (*n* = 5) or shCTNNB1 (*n* = 5) SKMEL28 aggregates were injected into the IV^th^ ventricle of the stage 12/13 HH chick embryo, and analyzed after 96 h of further incubation as above. SKMEL28 control shRNA cells formed tumor nodules in the roof plate and neighboring host tissues with single infiltrating cells resembling the sprouting cells in (**a**). Knock-down of β-catenin via shRNA (shCTNNB1) completely blocked invasion of melanoma cells in the rhombencephalon. **c** BLM cells were used in the CAM metastasis assay. Pre-exposure of the BLM cells with PKF115–584 (0.5 μM for 2 h) significantly reduced liver micrometastasis but showed no effect on primary tumor formation on the CAM (Mann-Whitney test)

To test whether the infiltration of single melanoma cells from aggregates observed in the neural crest or the rhombencephalon could be reproduced in a similar 3D–environment in vitro, we embedded SKMEL28 melanoma cell aggregates into a collagen matrix with or without 3T3 cells or Wnt3a secreting 3T3-Wnt3a cells as stroma. Sprouting (invasion) of SKMEL28 cells was radially measured (*n* = 6 per treatment group) at 0 h and 48 h. Stromal cells (3T3) increased sprouting of SKMEL28 cells, and this effect was significantly more pronounced in the 3T3-Wnt3a stromal environment (Fig. 8a), thus confirming the in vivo results described above.

To validate our findings with BLM melanoma cells in the chick brain, we conducted a confirmation experiment using SKMEL28 melanoma cells with a stable knock-down of β-catenin via shRNA [17]. As expected, in control shRNA SKMEL28 cells, tumor nodules formed in the roof plate of the rhombencephalon, and single cells infiltrated the neighboring host tissues (Fig. 8b and c; compare Fig. 7a and sprouting in Fig. 8a). The β-catenin shRNA SKMEL28 cells showed a different result: Here, the tumor cells had formed aggregates attached to the roof plate, without infiltration of the host tissue (Fig. 8d and e), thus corroborating the results obtained with PKF115–584.

Besides invasion, metastasis comprises several additional steps like intra- and extravasation. Therefore, we performed an *in ovo* chorioallantoic membrane (CAM) metastasis assay using chick embryos. Control BLM cells or PKF115–584 pretreated (0.5 μM for 2 h) BLM cells (1 × 10^6^ cells in 10 μl) were seeded onto the CAM of 8 day chick embryos and further incubated for 7 days. Then the upper CAMs containing the melanoma tumors and the livers with putative micrometastases were harvested and analyzed by qPCR for human DNA. Whereas the short pre-treatment with PKF115–584 did not significantly affect the initiation and growth of tumors at the side of inoculation (*p* = 0.8, Mann-Whitney test), the number of micrometastatic cells in the livers of chick embryos in the PKF115–584 group was significantly reduced (*p* = 0.002, Mann-Whitney test). This strongly highlights the importance of Wnt−/β-catenin signaling during metastasis of melanoma cells.

## Discussion

In the present study, we demonstrate a novel role of Wnt3a and the β-catenin signaling pathway in neural crest migration and malignant invasion of human melanoma cells.

Current therapeutic strategies for the treatment of metastatic melanoma focus on two major approaches with proven clinical efficacy: (i) direct targeting of activated oncogenes in melanoma cells such as BRAF [53] or (ii) indirect targeting of melanoma cells by T-cell stimulation with anti-CTLA4- or anti-PD-1-antibodies [54, 55]. Although these therapies caused a paradigm shift and were able to improve the 3-years overall survival of patients diagnosed with metastatic melanoma between 2011 and 2014 to 23% [56], both approaches bear major drawbacks, which are reflected by the limited duration of the initial clinical response. Only a subpopulation of melanomas harbors the crucial oncogenic BRAF-mutation, and even in mutated melanomas a therapy resistance rapidly develops [57]. We have recently shown that β-catenin is one potent mediator of resistance towards BRAF inhibition [46]. In line, high levels of ZEB1 expression (an EMT inducer) are associated with inherent resistance to MAPKi in BRAFV600-mutated cell lines and tumors [58]. Likewise, only a half of the patients clinically responds to T-cell stimulation, which is at least partially due to the fact that cytotoxic CD8^+^ T-cells only recognize major histocompatibility complex (MHC) class I (MHC-I)-expressing melanoma cells. However, the alteration of MHC-I expression together with an impaired response to interferons is a frequent event during cancer (and melanoma) progression, allowing cancer cells to evade the endogenous or therapeutic immunosurveillance [59]. A second plausible explanation for resistance to the novel immunotherapies might be the tumor-intrinsic oncogenic signals such as active β-catenin signaling, that mediate T-cell exclusion at the site of the tumor and thus resistance to anti-PD-L1/anti-CTLA-4 therapy [38, 60]. Such mechanisms might be reflected by the association of WNT3A expression and melanoma patient survival which we have elaborated in this project. Therefore, additional and fundamentally different therapeutic approaches are still desperately needed to improve therapies and finally overall- and long-term survival of advanced melanoma patients.

Our approach is to draw an analogy between embryonic growth and cancer growth. In particular, neural crest signaling pathways seem to be a promising target for the inhibition of melanoma cell invasion and metastasis [14]. Therefore, in the current study we first addressed the spatial expression of β-catenin in primary human melanomas. Interestingly, we found that β-catenin was predominantly expressed in melanoma cells of the invasive front with a spindle-like morphology. Therefore, we hypothesized that β-catenin-inhibition could affect melanoma cell migration and invasion in the neural crest. In the embryo, emigration of neural crest cells from the neural tube is designated as EMT. EMT represents a complex change in cell morphology and migratory potential of embryonic cells and is induced in the embryo mainly by BMPs and Wnt-signaling [1–4], and vice versa inhibited by their antagonists. EMT comprises two consecutive steps [61, 62]: (i) the neural crest compartment is induced in the epithelium of the neural tube, which is morphologically characterized by the disintegration of the basal lamina in the region of the lateral roof plate. (ii) Neural crest cells are induced to start migration from the dorsal edges of the neural tube along their designated medial and lateral pathways. Hence, EMT (governing embryonic neural crest migration and possibly melanoma cell invasion in the patient) of melanoma cells as neural crest descendants should be analyzed in the neural crest environment.

To verify our analogy hypothesis, we therefore used our chick embryo model in two different experimental settings: First, we injected human melanoma cells into the lumen of the neural tube of stage 12/13 HH chick embryos to analyze their capacity for spontaneous neural crest migration. Before injection, the melanoma cells were pre-conditioned with either the agonist Wnt3a or with the β-catenin-inhibitor PKF115–584. Interestingly, the agonist and the antagonist had opposing impacts on melanoma cell behavior in the neural crest compartment: Wnt3a enhanced, and PKF115–584 abrogated the spontaneous neural crest migration of SKMEL28 cells in vivo, which is in line with the effects of the neural crest-inducer BMP-2 and its physiological antagonist noggin [13]. The impact of Wnt-signaling on melanoma cell migration and EMT (reflected by changes in aggregation and migration) could be reproduced in vitro. This finding is challenged by several publications that rather show the opposite effects [26–29]. However, a recent study clearly showed that canonical Wnt signaling is a pro-invasive factor in the context of active PI3K signaling pathway due to PTEN deficiency [63]. This effect was found to be mediated by context dependent regulation of catabolic processes by β-catenin. Since in our cell culture models the PI3K signaling pathway is activated (high phosphorylation of Ser473 of AKT) we believe that our models reflect this situation. This is further substantiated by the bad impact of low PTEN expression levels on melanoma patient survival in the context of a high expression of WNT3A (whereas PTEN levels have no impact on survival for WNT3A low melanomas).

RNA expression analyses of melanoma cells pre-conditioned with either Wnt3a or PKF115–584, and of zebrafish embryo neural crest cells demonstrated overlapping genes. Using siRNA mediated down-regulation, we could functionally demonstrate an impact on EMT of the overlapping genes in melanoma cells in vitro, thus corroborating the RNA-expression results. In this respect, we were able to identify inhibin beta A as novel, β-catenin-regulated candidate gene that hinders melanoma cell invasion. Interestingly, by screening melanoma data from The Cancer Genome Atlas, we discovered a correlation of high expression of CYR61 or (by trend) INHBA with increased overall survival of melanoma patients, clearly underlining the clinical significance of our findings. This is in line with a previous report showing that Cyr61 expression in melanoma cells reduces tumor growth and metastasis [64].

Since we also detected MITF and its target gene tyrosinase (TYR) to be regulated upon modification of canonical Wnt signaling in SKMEL28 melanoma cells it could be that MITF is at least partially involved in the pro-migratory effects in vitro and in vivo. This would support a recent publication, which found that MITF is required for melanoma cell migration and invasion independent of BRN2, meaning in MITF^high^ and MITF^low^ melanoma cells [65]. Again, this is a topic full of conflicting data which is mirrored by the complexity of the MITF rheostat model [66]. For example, Fane et al. could recently show that MITF and BRN2 are inversely correlated and BRN2 expression drives melanoma cell migration and invasion via the nuclear factor IB [67]. Since we did not see strong effects on MITF gene expression in all cell lines tested, we do not assume MITF to play a leading part in the observed effects. TYR was additionally identified as a direct target of LEF1 dependent gene transcription [68].

The question arose, if the physiological neural crest migration of melanoma cells was equal to malignant invasive migration, as encountered in advanced melanoma patients, and if such malignant invasion was also accessible for pharmacological manipulation through inhibition of endogenous Wnt-signaling. Thus, we injected untreated or PKF115–584 pre-conditioned human melanoma cells in the rhombencephalon of the chick embryo and analyzed malignant invasion after 4 days. As expected, the untreated melanoma cells formed tumor nodules in the roof plate and adjacent mesenchyme, with single cells and streets of cells invading the chick host tissues and blood vessels. Interestingly, only actively invading melanoma cells with a mesenchymal morphology had a prominent cytoplasmic β-catenin expression, and not the cells in the tumor nodule displaying a compact, epithelial-like morphology. This was in line with melanoma cells in primary melanomas displaying a high cytoplasmic β-catenin expression at the invasive front. To our surprise, the PKF115–584 pre-conditioned melanoma cells had undergone apoptosis, and in none of the embryos of this cohort a viable tumor nodule could be detected. This is particularly interesting since 48 h after injection into the neural tube, even PKF115–584 pre-conditioned melanoma cells were still positive for the proliferation marker MIB1, and suggests that inhibition of Wnt-signaling with PKF115–584 might lead to both complete inhibition of neural crest migration and invasion (after 48 h) and to a delayed induction of cell death (up to 96 h). This is in line with previous reports showing both anti-proliferative and anti-invasive effects of β-catenin-inhibition in melanoma cells [16, 17, 30].

## Conclusions

Together, our results show a novel role of Wnt3a/β-catenin signaling in melanoma cell neural crest like migration and invasion and suggest the participation of β-catenin during active melanoma cell invasion into the dermal matrix, the brain microcompartment and during metastasis to the liver. The crucial role of the embryonic neural crest signaling for melanoma progression is underlined by a neural crest related gene signature. Since non-canonical Wnt signaling pathways that frequently involve Wnt5a are complementary strong contributors to the invasive behavior of melanoma cells, we believe in a context dependent, plastic effect of distinct Wnt ligands and their corresponding receptorsor adaptors. We therefore suggest that the malignant, invasive growth of melanoma cells could be inhibited by novel inhibitors of Wnt secretion as an additional therapeutic approach that might prevent metastasis in melanoma patients.

## Additional files

Additional file 1: **Figure S1.** Low expression of PTEN worsens the survival of patients with WNT3A^high^ or WNT3^high^ melanomas. **Figure S2.** Wnt3a enhances and PKF115–584 blocks neural crest migration of SKMEL28 melanoma cells in the chick embryo in vivo*.* **Figure S3.** Wnt3a induces and PKF115–584 inhibits migration and typical features of EMT of melanoma cells in vitro*.* **Figure S4.** Wnt3a induces invasive growth of melanoma cells in organotypic tissue skin reconstructs (TSR). **Figure S5.** Gene expression during neural crest induction in the zebrafish embryo and upon Wnt stimulation in SKMEL28 melanoma cells. (PDF 1054 kb)

Additional file 2: **Table S1.** List of 44 differentially expressed genes in human SKMEL28 melanoma cells (FC: fold change treatments vs. control, left columns) compared to the gene expression data of MLANA^high^ versus MLANA^low^ melanoma samples from the TCGA database (right columns). (XLSX 53 kb)
