# Supplementary material for: Wnt-signaling enhances neural crest migration of melanoma cells and induces an invasive phenotype
Source: Mol Cancer. 2018 Feb 17;17:59. doi: 10.1186/s12943-018-0773-5 (PMC5816360; doi:10.1186/s12943-018-0773-5)
Supplement: Supplementary file 1 — Figure S1. Low expression of PTEN worsens the survival of patients with WNT3Ahigh or WNT3high melanomas. Figure S2. Wnt3a enhances and PKF115–584 blocks neural crest migration of SKMEL28 melanoma cells in the chick embryo in vivo. Figure S3. Wnt3a induces and PKF115–584 inhibits migration and typical features of EMT of melanoma cells in vitro. Figure S4. Wnt3a induces invasive growth of melanoma cells in organotypic tissue skin reconstructs (TSR). Figure S5. Gene expression during neural crest induction in the zebrafish embryo and upon Wnt stimulation in SKMEL28 melanoma cells. (PDF 1054 kb) [file 12943_2018_773_MOESM1_ESM.pdf]

Supplementary Material:

Figures S1-S5

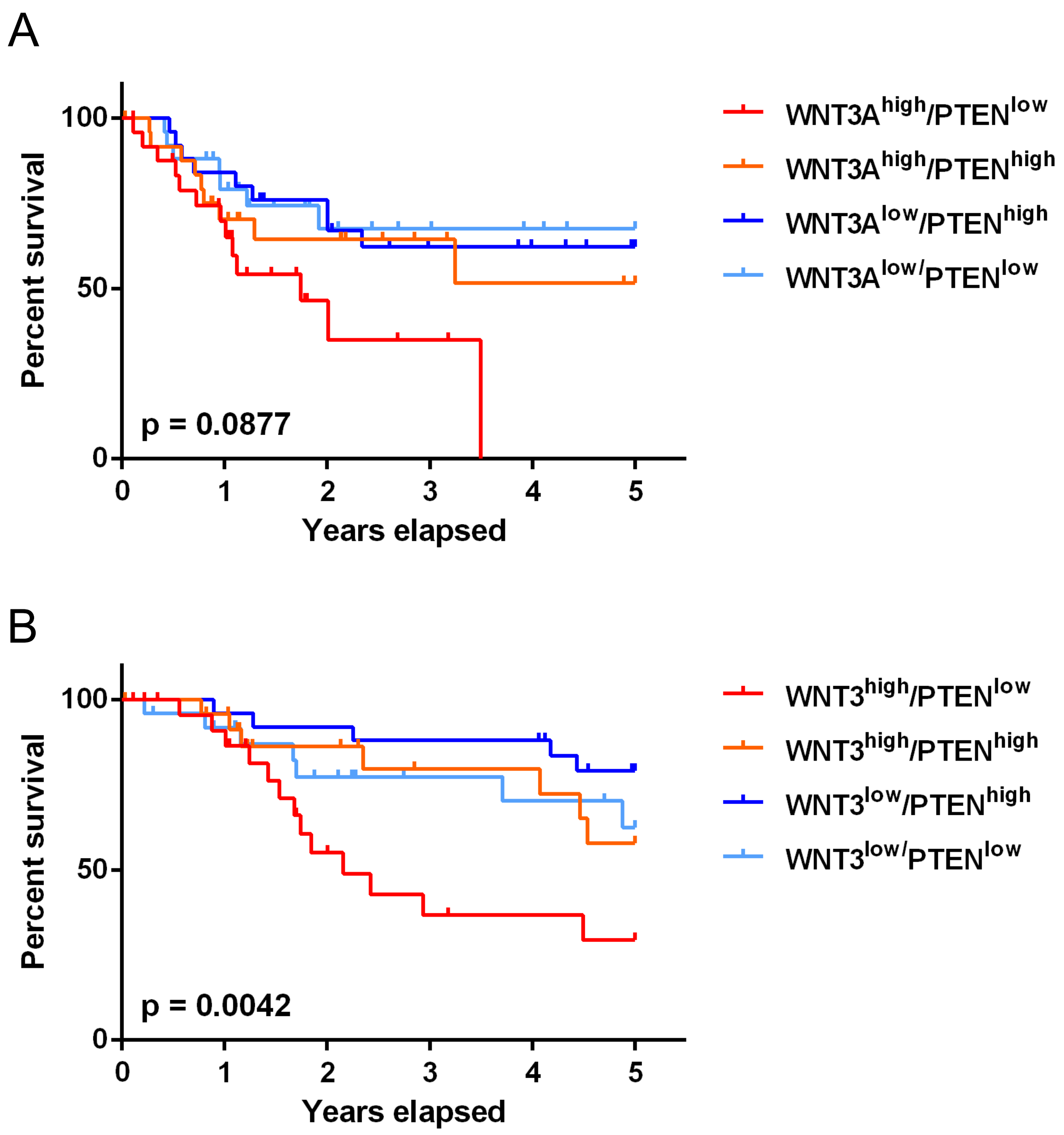

Fig. S1: Low expression of PTEN worsens the survival of patients with WNT3A^{high} or WNT3^{high} melanomas

A subgroup-analysis of the TCGA melanoma data set for the top and bottom (n=50 each) WNT3A (A) or WNT3 (B) melanomas reveals a significant impact of PTEN expression levels on the survival of the patients. Low PTEN expression significantly ($p = 0.0042$) deteriorates survival of the patients with melanomas with high WNT3 expression levels ($p = 0.0877$ for WNT3A). PTEN seems not to impact survival of the other (WNT3^{low}/WNT3A^{low}) patient groups.

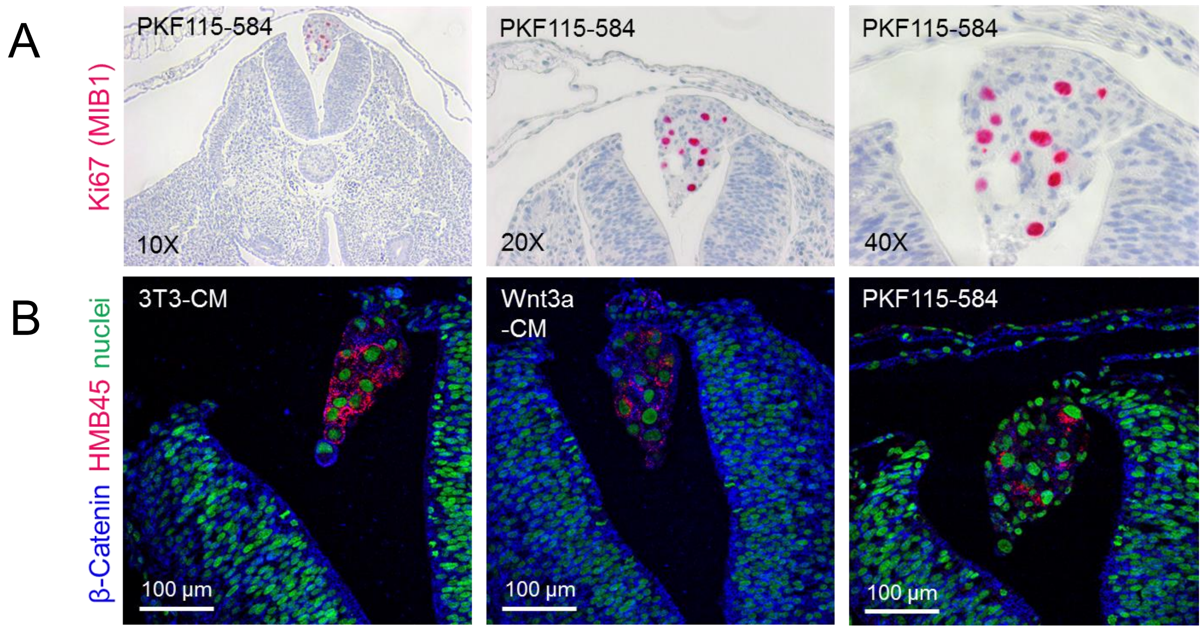

Fig. S2: Wnt3a enhances and PKF115-584 blocks neural crest migration of SKMEL28 melanoma cells in the chick embryo *in vivo*

A: Anti-MIB1 (Ki67) immunohistochemistry (red nuclear staining) confirmed the proliferative activity and viability of the transplanted PFK115-584 pre-conditioned melanoma cells.

B: IF analysis of the chick embryos using anti-β-catenin (blue) showed that the PKF115-584 pre-conditioned cells showed a strongly reduced cytoplasmic expression of β-catenin in the HMB45 positive (red) melanoma cells. Nuclei were stained with YO-PRO-1 and are depicted in green.

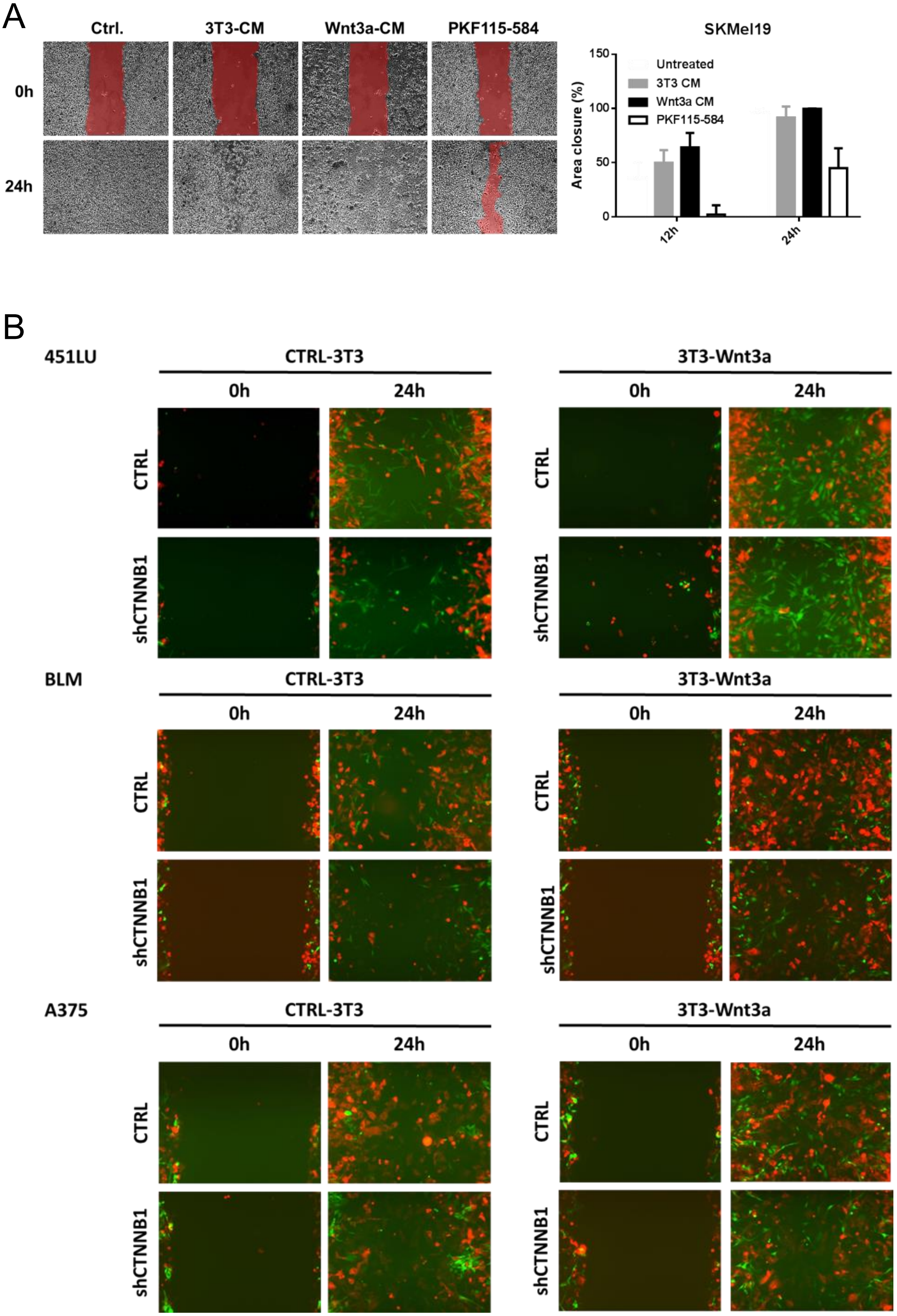
A
Ctrl.
3T3-CM
Wnt3a-CM
PKF115-584
0h
24h
SKMel19
Area closure (%)
150
100
50
0
Untreated
3T3 CM
Wnt3a CM
PKF115-584
12h
24h
B
451LU
CTRL-3T3
3T3-Wnt3a
0h
24h
CTRL
shCTNNB1
BLM
CTRL-3T3
3T3-Wnt3a
0h
24h
CTRL
shCTNNB1
A375
CTRL-3T3
3T3-Wnt3a
0h
24h
CTRL
shCTNNB1

C

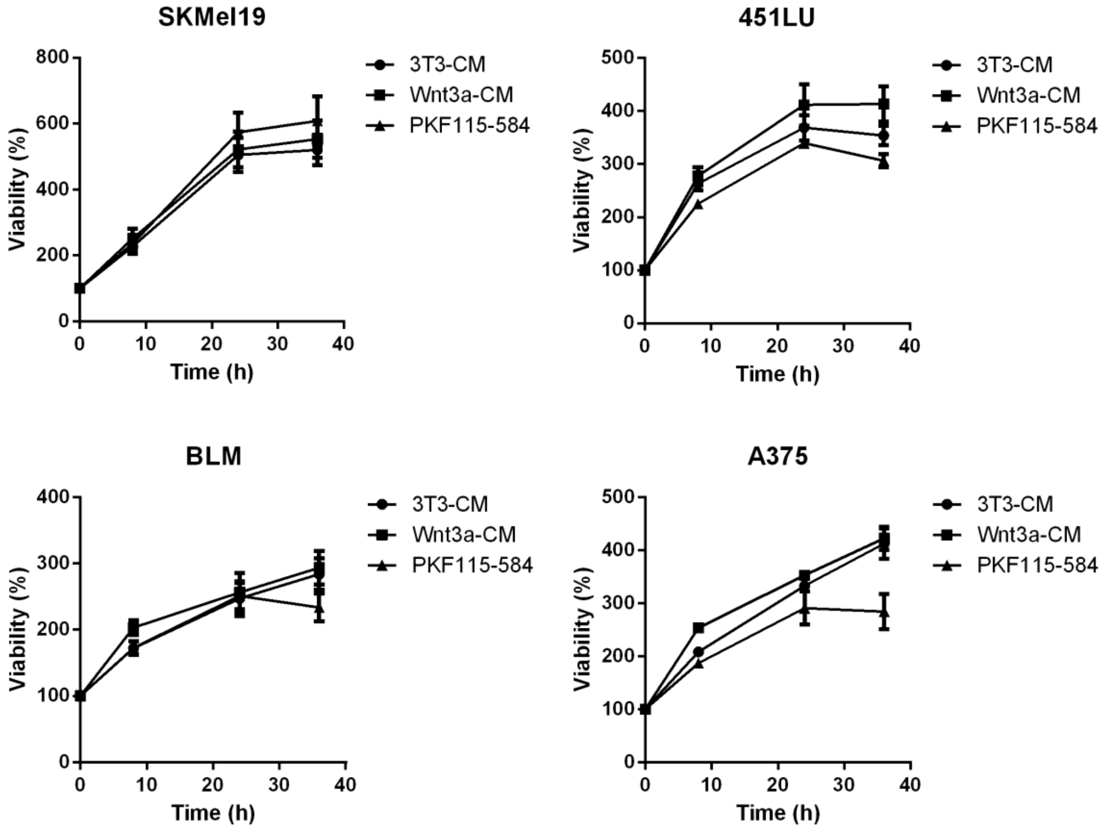

Fig. S3: Wnt3a induces and PKF115-584 inhibits migration and typical features of EMT of melanoma cells *in vitro*

A: Scratch assay of human SKMEL19 melanoma cells showing by trend an increased migration upon stimulation with Wnt3a conditioned medium (Wnt3a CM) and decreased migration upon treatment with PKF115-584) when compared to untreated and 3T3-medium conditioned cells (3T3-CM).

B: 3T3 control fibroblasts (CTRL-3T3) or Wnt3a secreting 3T3 fibroblasts (3T3-Wnt3a) were labeled with CFSE and prepared to set up co-cultures with DsRed-labeled melanoma cell lines (451Lu, BLM or A375) in a 1:4 ratio before scratching. Migration of the cells into the wound was photographed at 24 h post scratching. Small-hairpin RNA specific for CTNNB1 was lentivirally transduced into the cell lines to test the

dependence of migration on β-catenin. In 3T3-Wnt3a containing samples more DsRed positive melanoma cells migrated into the gap and knockdown of β-catenin reduced this effect. 3T3-Wnt3a fibroblasts showed increased migratory potential compared to control 3T3 cells.

C: A real-time RealTime-Glo™ MT Cell Viability Assay was performed to investigate the impact of the different treatments on the cell viability of the melanoma cell lines. 2,500 melanoma cells were seeded into 96well plates. 0.5 µM PKF115-584 was used for a 4 h incubation before the start of the assay.

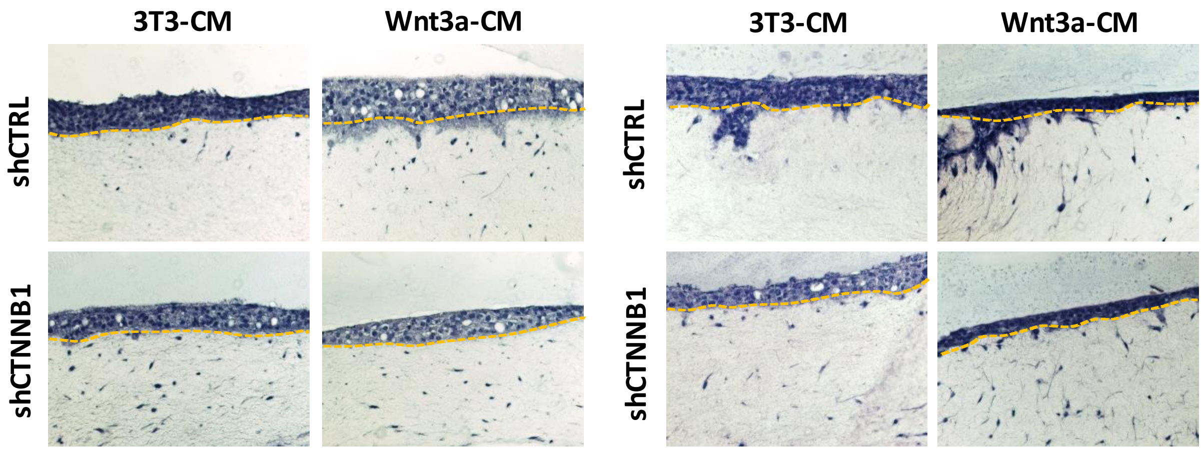

Fig. S4: Wnt3a induces invasive growth of melanoma cells in organotypic tissue skin reconstructs (TSR)

TSR were prepared in 24 well plates with primary human fibroblasts (150.000 cells) and collagen I (1.35 mg/ml) and HaCat cells (100,000) as epidermal layer. Tumor cells (25,000) were seeded together with the HaCat cells to test the invasive capacity of the malignant cells. Human melanoma cell lines (left group: BLM, right group: A375) exposed to Wnt3a conditioned medium (Wnt3a-CM) show a pronounced invasive phenotype into the dermal part when compared to cells exposed to control medium (3T3-CM). After knockdown of β-catenin using lentiviral-transduced shCTNNB1 cells no cells entering the dermal part of the TSR could be identified.

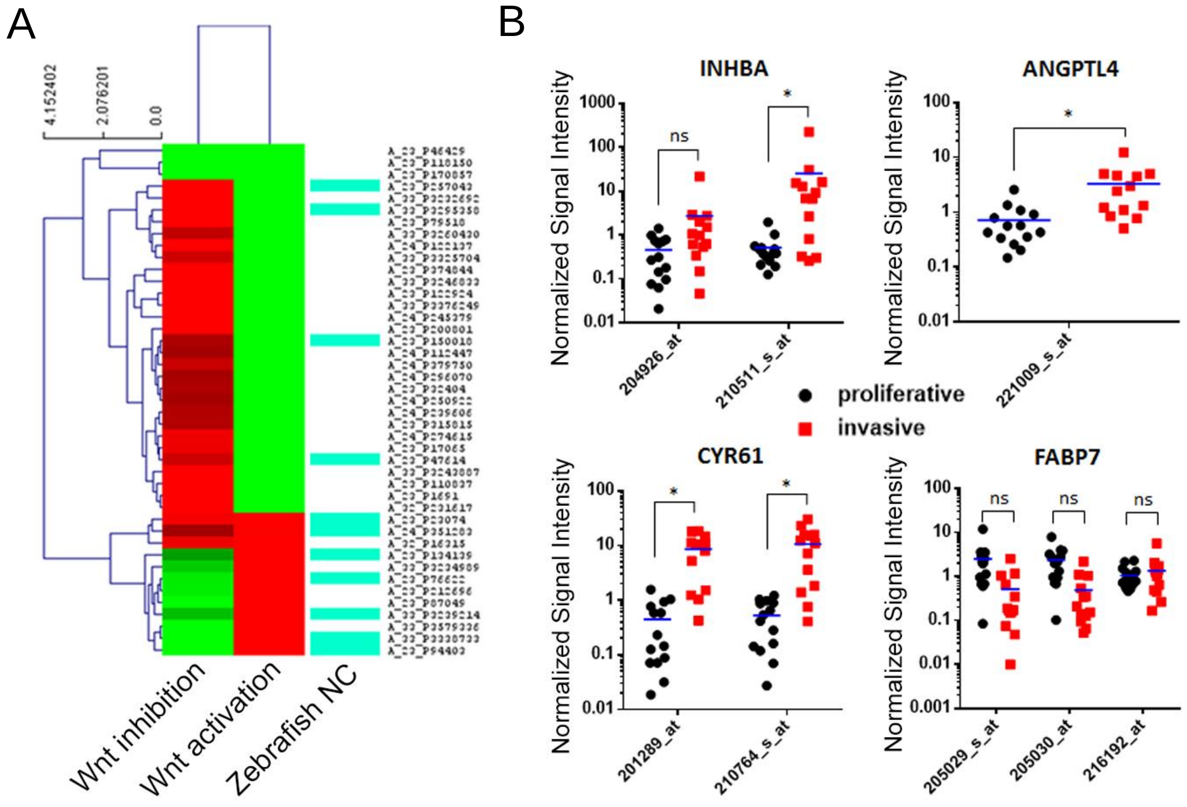

Fig. S5: Gene expression during neural crest induction in the zebrafish embryo and upon Wnt stimulation in SKMEL28 melanoma cells

A: mRNA expression of SKMEL28 cells (untreated, Wnt3a- or PKF115-584-stimulated (0.5 µM)) was compared to mRNA expression of the zebrafish embryo neural crest. 44 genes were differentially expressed after Wnt induction (Wnt3a) or Wnt inhibition (PKF115-584) of SKMEL28 cells (refer to Table 1). Six overlapping genes (GEM, ANGPTL4, DUSP5, PHLDA2, IFI44, and FABP7) were found by comparing the 44 differentially expressed genes in SKMEL28 cells to the gene expression of the zebrafish neural crest.

B: Expression levels of INHBA, ANGPTL4, CYR61, and FABP7 in melanoma cells with a distinct proliferative or invasive phenotype. Different gene specific probes are shown (*: $p<0.05$, One way ANOVA).
